# A Comparison between Chocolate Milk and a Raw Milk Honey Solution’s Influence on Delayed Onset of Muscle Soreness

**DOI:** 10.3390/sports4010018

**Published:** 2016-03-07

**Authors:** Andrew Hatchett, Christopher Berry, Claudia Oliva, Douglas Wiley, Jacob St. Hilaire, Alex LaRochelle

**Affiliations:** Health Sciences, Department of Biology, Franklin Pierce University, Rindge, NH 03461, USA; Berryc11@live.franklinpierce.edu (C.B.); Olivac12@live.franklinpierce.edu (C.O.); drwilley@email.uark.edu (D.W.); St.Hilairej12@live.franklinpierce.edu (J.S.H.); Larochellea12@live.franklinpierce.edu (A.L.)

**Keywords:** DOMS, chocolate, milk, raw, recovery, soreness, honey, strength, exercise

## Abstract

This investigation sought to examine the effect that a chocolate milk solution (CMS) and a raw milk solution (RMS) had on lower extremity induced delayed onset of muscle soreness (DOMS). Twenty trained male participants completed a set of questionnaires, prior to completing a lower extremity DOMS protocol, to determine the level of discomfort and functional limitations. Once the DOMS protocol was completed, participants were randomly assigned to either the CM or RM group. Once assigned, participants ingested 240 mL of the respective solution and completed the same set of questionnaires immediately post, 24-, 48- and 72-h post DOMS protocol. Additionally, for 10 days post-ingestion participants were contacted to learn if any negative effects were experienced as a result of ingesting either solution. Both groups reported an increase in lower extremity discomfort at each data collection interval post-DOMS protocol (post, 24-, 48- and 72-h). Participants assigned to the RM group reported high discomfort post and a relative decline in discomfort from immediately post-DOMS protocol to 72-h post. The RMS group reported substantially less discomfort at 72-h when compared to the CMS group. Ingestion of a raw milk solution immediately post strength exercise can substantially reduce the level of self-reported discomfort associated with DOMS.

## 1. Introduction

Delayed onset of muscle soreness (DOMS) is an outcome often associated with exercise-induced muscle damage (EIMD) [1]. DOMS usually occurs after an increase in intensity or volume of training or when the schedule of exercise is altered [2,3,4]. The classical symptoms of DOMS include strength losses, pain, swelling, tenderness or stiffness, loss of full range of motion, flexibility, force production and mobility [2,5,6]. It is known that delayed onset muscle soreness increases the intensity of pain within 24 h after exercise, reaches a peak between 24 h and 48 h, disappears within 5–7 days after exercise [7]. A number of modalities have been investigated in the search for a treatment that may reduce the effects of EIMD and/or accelerate recovery [1,8].

A typical method athletes and active persons attempt to aid this process is through the use of dietary supplementation via post-exercise protein drinks [9]. Chocolate milk has also been observed to be an effective recovery aid after exercise. For instance, Karp *et al.* [10] observed similar increases in time to exhaustion and total work for individuals that consumed chocolate milk when compared to a traditional electrolyte replenishing drink subsequent to exhaustive exercise [9]. Evidence continues to emerge in support of chocolate milk, a cocoa-based beverage, as an effective recovery aid due to its optimal carbohydrate to protein ratio [10,11,12,13,14,15].

Heat treatments, such as those used in pasteurization, are generally assumed to impair protein quality [16]. The cocoa found in chocolate milk has gone through the alkalization process therefore reducing the antioxidant potential [11,17]. Recently, unpasteurized raw milk consumption has increased in popularity and emerged into a nationwide movement despite the acknowledgement of risks associated with foodborne pathogens [18,19]. Raw milk, or unpasteurized milk, is fresh and has not undergone heat treatment (pasteurization). Proponents of raw milk believe that due to the fact it has not been pasteurized, it contains greater amounts of and higher quality nutrients, enzymes and bacteria [20]. Honey has been used for its healing, nutritional and therapeutic properties since ancient times. It possesses anti-bacterial, anti-inflammatory and anti-oxidant properties that may be beneficial for preventing inflammatory processes [21]. Perhaps due to the controversial nature of raw milk, limited research has been conducted with raw milk.

With chocolate milk being a processed food commonly used and researched as a recovery aid from intense physical activity and raw milk being a relatively novel product, especially when combined with honey for the same purpose a comparison between the two has yet to be conducted. Therefore, this study examined the effects of a chocolate milk solution (CMS) and a raw milk and honey solution (RMS) on delayed onset muscle soreness.

## 2. Materials and Method

### 2.1. Study Design

This study was designed to investigate if participants reported a difference in delayed onset of muscle soreness after ingesting a chocolate milk solution or a raw milk and honey solution. Participants reported to the Functional Movement Laboratory (FML) at Franklin Pierce University on six different occasions (T1–T6) during the study. T1 consisted of a study briefing designed to inform the participants of the study protocol, obtain completed informed consent agreements, record anthropomorphic measurement and to establish a one repetition maximal load (1RM) of a barbell back squat. Seventy-two hours post-T1 participants reported to the FML for T2. T2 consisted of participants completing the DOMS measure, followed by the completion of the DOMS protocol. T3 occurred immediately after the participant completed the DOMS protocol, where the randomly assigned milk solution was offered and the DOMS measure was completed. Each of the following data collection periods the DOMS measure was completed: T4 (24 h post), T5 (48 h post) and T6 (72 h post). Following the T6 face-to-face data collection each participant was contacted each day for seven days to determine if they were experiencing any abnormal effects due to their participation in the study. This study was approved by the Franklin Pierce University Rindge Institutional Review Board Sub-Committee.

### 2.2. Participants

Twenty healthy male collegiate sprint football players participated in this study. Subjects had a mean age of 20.00 ± 0.85 years. (mean ± SD), a height 174.69 ± 1.68 cm, a mean weight of 79.37 ± 1.52 kg, a mean 1RM back squat of 126.55 ± 3.62 kg and a mean 80% of their individual 1RM back squat of 102.51 ± 2.77. All subjects were informed of the study protocol and potential negative side effects associated with participation prior to providing their written consent.

### 2.3. Experimental Trial

Participants reported to the FML on six different occasions (T1–T6) during the study. T1 consisted of a study briefing designed to inform the participants of the study protocol, obtain completed informed consent agreements, record anthropomorphic measurements and to establish a one repetition maximal load (1RM) of a barbell back squat. During this initial meeting it was stressed to the participants the unique nature of the study and the potential side effects associated with the ingestion of raw milk. The participants were also offered detailed information regarding raw milk both verbally and in hardcopy form. It was further stressed to the participants that once they have listened to and read the information regarding raw milk that their participation was completely voluntary and they could withdraw at any time during the study with no negative consequences.

Seventy-two hours post-T1 participants reported to the FML for T2. T2 consisted of participants completing the measure of DOMS. The measure was a FPS-R (Face Pain Scale—Revised) prior to the next phase of the study to determine level of lower extremity discomfort prior to participating in the second phase of T2. The second phase of T2 involved the participants completing four sets of 10 repetitions of 80% of their previously obtained 1RM after a sufficient warm up. Participants were monitored by trained research assistants for complete range of motion, accuracy of amount of weight lifted, and accuracy of repetitions completed, as well as for safety of the participant. Once the participant completed the fourth set of 10 repetitions, they were guided to another member of the research team to be randomly assigned to either the chocolate milk (CM) group or the raw milk (RM) group.

When group assignment was made the participant completed the DOMS questionnaire. Once the questionnaire was completed, the participant was offered approximately 240 mL of either beverage and asked to consume the beverage promptly (within 5 min post DOMS protocol completion). Participants were then asked to report to the FML 24 (T4), 48 (T5) and 72 (T6) hours post T3 for completion of the FPS-R.

Additionally, a member of the research team contacted each participant in order to determine if they were experiencing any internal discomfort attributed to the ingestion of either beverage immediately after consumption and once per day for10 days following consumption. If any ill effects were reported a negative side effect protocol was prepared for implementation.

### 2.4. DOMS Measures

Delayed onset of muscle soreness (DOMS) was assessed with two independent instruments. One instrument was a visual analog scale.

FRP-S: Participant’s perceived pain discomfort level was assessed using a standardized protocol. Assessments of discomfort were performed with a blank FRP-S for each assessment to minimize bias from previous reporting. The FPS-R is a 6-point scale, with 6 different faces that represent increasing levels of pain intensity. Participants were asked to select the one expression that best characterized his pain intensity, from the left-most face (“No pain”), to the right-most face (“Very much pain”). Each illustration corresponded to a numeric score (0, 2, 4, 6, 8, or 10). Research supports the validity of this pain measure [22].

### 2.5. Statistical Analysis

To calculate the chances of benefit and harm, the smallest worthwhile or important effect for each dependent variable was the smallest standardized (Cohen) change in the mean −0.2 times the between subject SD for baseline values of all participants [23]—which has been used in similar investigations [23]. Practical inferences were drawn using the approach identified by Hopkins (2006) and utilized by Cockburn, 2010. Quantitative chances of benefit and harm were assessed qualitatively: <1% indicated almost certainly none; 1% to 5% indicated very unlikely; 5% to 25% indicated unlikely; 25% to 75% indicated possibly; 75% to 95% indicated likely; 95% to 99% indicated very likely; and >99% indicated almost certainly [23,24]. This method provides a way to qualify clear outcomes with a descriptor that represents the likelihood that the true value will have the observed magnitude. They are also free of the burden of Type I and Type II errors because they are probabilistic rather than definitive statements [24] Because of the large number of comparisons that could be reported, we only reported changes from baseline to 24, 48 and 72 h. Previous research has shown that the point at which milk-based carbohydrate-protein supplements become beneficial is 48 h after muscle-damaging exercise [25].

## 3. Results

Physical characteristics of both groups were similar. The mean age of participants was 20 years, mean height 68.775 inches and mean weight 175.89 pounds. Also similar between each group were the strength measures: mean 1RM back squat was 279 pounds and the mean value for 80% of each respective 1RM back squat was 226 pounds. (Table 1).

Both groups showed an increase in DOMS as a result of the assigned and completed protocol. Reported lower extremity soreness peaked at 48 h for the CM group and immediately after completing the DOMS protocol for the RM group. Both groups reported similar levels of discomfort 24 h post DOMS protocol with an increase in discomfort from 24 to 48 h post DOMS protocol.

At 72 h post-DOMS protocol, both groups reported a lower level of discomfort trending to baseline values.

**Figure 1:**
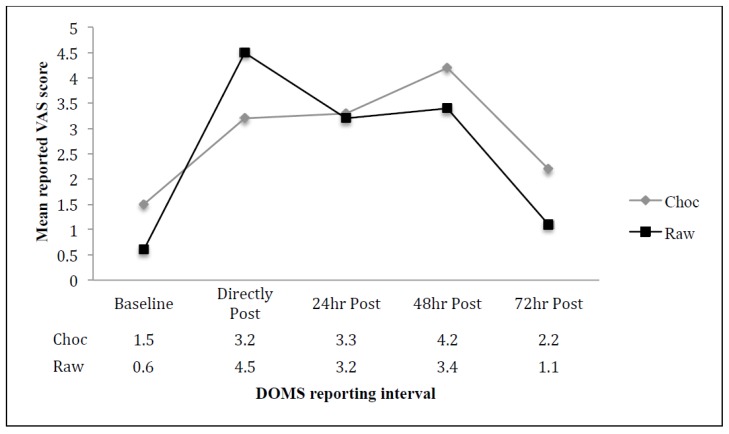
Changes in reported visual acuity scale (VAS) score regarding lower extremity muscle soreness pre-exercise induced muscle soreness (EIMS) protocol, directly post EIMS, 24 h, 48 h and 72 h post-EIMS for both the Raw Milk Solution (RMS) group and the Chocolate Milk Solution (CMS) group.

Each dependent variable was analyzed, using a published spreadsheet [23], to determine the effect of the respective solution as the difference in the change between each group. Participant descriptive data and muscle soreness data are presented as absolute means + SD (Figure 1).

There was a likely benefit to the ingestion of the RMS at 48 (0.8 ± 2.55) and 72 h (1.1 ± 1.68) post-EIMS protocol. The difference between the CMS group and the RMS was unclear at baseline (0.1 ± 1.35) and 24 h post-EIMS (0.1 ± 1.69) (Table 2).

## 4. Discussion

This study investigated how a raw milk and honey solution would compare to a chocolate milk solution in the attenuation of lower extremity DOMS. The primary finding of this study was that the consumption of a raw milk and honey solution immediately following relatively aggressive lower extremity exercise reduced reported muscle soreness to a greater extent when compared to chocolate milk over a 72-h time frame. Secondary findings indicate that the consumption of a raw milk and honey solution elicited no reported negative side effects up to 10 days post consumption.

Participants for this study were members of a small University’s Sprint Football team. This particular group was chosen to participate due to the fact that the members of this team had recently (within the past seven days) finished their spring training cycle in which each of the athletes completed the same program during this cycle allowing for a very similar training state among participants. Additionally, the Sprint Football is unique when compared to traditional football in that the athletes must maintain a body weight of 172 pounds and a minimum of 5% body fat during the football season [24]. This requirement has lead to the athletes being of very similar size and capacity.

Delayed Onset of Muscle Soreness and milk products.

The reasons behind DOMS have been a steady interest for many sports scientists for a long time. Although several factors including lactic acid, connective tissue damage surrounding muscles, muscle temperature, muscle spasm, inflammatory responses, free radicals, and nitric oxides have been suggested for causing DOMS, there is no clear explanation [26,27,28]. Previous literature has speculated that the cause of DOMS is due to structural muscle damages and perturbation of calcium homeostasis or acute inflammatory responses to exercise [28,29,30].

Consistent with previously reported results, the consumption of a milk-based solution with an elevated carbohydrate level reduces the DOMS [23]. However, what has not been reported prior to this study is the difference between a raw milk solution and a commercially available milk solution with elevated carbohydrate levels. Advocates for raw milk have made the case that the pasteurization process denatures the macronutrients and many of the micronutrients present in the milk.

In a recent review of nutritional interventions on delayed onset muscle soreness Kim and Lee (2014) presents information regarding the influence of polyphenol. The major biological functions of polyphenol are antioxidant capacity and anti-inflammation. According to previous studies, a potential mechanism for reducing DOMS by ingestion with polyphenol is its action on membrane stability and reduced lipid peroxidation by inhibiting peroxyl radical activation [28]. Honey contains a number of antioxidants from a variety of sources, including polyphenolics. The antioxidant components found in varieties of honey have been associated with improving vasodilation and conditions associated with inflammatory response [31]. 

In should be noted, the small independent dairy farm that the raw milk was obtained for this study undergoes laboratory testing to determine the safety of the milk it offers on a monthly basis. Prior to this research being conducted, the researches obtained a copy of the most recent laboratory results. The samples used in the laboratory tests were collected two days before the dairy collected the milk used in the study. Independent laboratory results indicated that the milk used in this study was safe for human consumption. The storage and distribution of the milk during this study was also consistent with appropriate food hygiene practices, thus limiting the opportunity for food-borne illness.

The exact mechanism for the greater decrease in DOMS when athletes consumed the RMS is unclear. Perhaps, as postulated by Cockburn *et al.* [23], this difference is due to limiting increases in protein degradation and stimulating protein synthesis of the polyphenol content of the honey used. Additionally, it may be possible that micronutrient denaturing through the process of pasteurization influenced the CMS. The fat content of the RMS was higher than that of the CMS, this may be associated with the difference in reported soreness. Further studies should expand on this research to explore different combinations of carbohydrate, milk type and fat content.

## 5. Conclusions

We have demonstrated that consuming a raw milk and honey solution immediately after muscle damaging exercise can increase the recovery rate substantially when compared to the consumption of chocolate milk, a commonly accepted recovery drink. Further research should be conducted examining the relationship between the unique nutrient combination found in a raw milk and honey solution.

## Figures and Tables

**Table 1:** Participant characteristics mean values (pooled and sorted by group assignment) *N* = 20 (Raw Milk Solution (RMS) *n* = 10, Chocolate Milk Solution (CMS) *n* = 10).

Variable	Pooled	RMS	CMS
Age (years)	20	19.9	20.1
Height (cm)	174.70	172.72	176.66
Weight (kg)	79.78	77.14	82.43
1RM (kg)	126.55	127.46	125.66
RM% (kg)	102.51	102.51	102.51

**Table 2:** Effect of solution timing on the changes between groups in muscle soreness following muscledamaging exercise.

Comparison	Mean Effect ± 90% CI	Qualitative Inference
Baseline	0.1 ± 1.35	Unclear
24 h	0.1 ± 1.69	Unclear
48 h	0.8 ± 2.55	Increase likely
72 h	1.1 ± 1.68	Increase likely
